# Clinical features predictive of vision loss in patients with vitreoretinal lymphoma: a single tertiary center experience

**DOI:** 10.1038/s41598-023-31414-0

**Published:** 2023-03-18

**Authors:** Mirinae Kim, Hyun Suh, Young Gun Park, Young-Hoon Park

**Affiliations:** 1 Department of Ophthalmology and Visual Science, College of Medicine, The Catholic University of Korea, 222 Banpo-daero, Seocho-Gu, Seoul, 06591 South Korea; 2 Seoul St. Mary’s Hospital, Seoul, South Korea

**Keywords:** Retinal diseases, Lymphoma

## Abstract

To clarify the long-term visual prognosis and prognostic factors for vision loss in patients with vitreoretinal lymphoma (VRL). This retrospective longitudinal study included 64 consecutive patients with VRL. We analyzed the best-corrected visual acuity (BCVA), optical coherence tomography findings, and clinical features at every visit. Significant vision loss was defined as a final BCVA ≥ 0.5 logMAR. Predictors of significant vision loss following treatment were evaluated using univariate and multivariate linear regression analyses. We included 113 eyes of 64 patients (mean age, 64.2 ± 10.9 years), and 49 patients (76.6%) showed bilateral ocular involvement. The mean follow-up duration was 35.4 ± 25.8 months. At diagnosis, 36 (56.3%), 17 (26.6%), and 11 (17.2%) patients had primary, secondary, and concurrent VRL, respectively. All eyes received intraocular methotrexate injections (mean, 17.1 ± 5.5 injections). The mean BCVA improved from 0.44 ± 0.28 at diagnosis to 0.33 ± 0.29 1 month after treatment initiation. Vision improved significantly after treatment (final mean BCVA, 0.24 ± 0.21). Univariate and multivariate analyses showed that baseline BCVA and retinal/subretinal infiltration were significantly correlated with vision loss. In this study, a good visual outcome was maintained for > 35 months in patients with VRL. Baseline BCVA and retinal/subretinal infiltration were significant predictors of vision loss after treatment for VRL.

## Introduction

Vitreoretinal lymphoma (VRL), previously known as primary intraocular lymphoma, is a rare intraocular malignancy that is a subtype of primary central nervous system lymphoma (PCNSL)^1,2^. VRL presents as high-grade B-cell lymphoma (diffuse large B-cell lymphoma) in > 95% of cases, and T-cell lymphoma is rare (< 5% of cases)^2–4^. VRL can present as an isolated disease or develop before, after, or concurrently with PCNSL^5,6^. Rates of non-ocular CNS involvement with VRL at diagnosis and over the course of the disease are 41% and 69%, respectively^6^.

The prognosis of VRL should be considered in two parts: survival and visual prognoses. Despite recent advancements in the diagnosis and treatment of VRL, the overall survival of patients with VRL remains poor. The overall survival period of isolated primary VRL is 31–58 months^7–10^, which is longer than that in concurrent VRL/ central nervous system (CNS) lymphoma (18–34 months)^7–9^. Because the long-term survival rate of the disease is significantly low, reports on long-term visual prognosis are lacking.

In recent years, diagnostic procedures and therapeutic options for VRL have advanced dramatically. However, studies on lymphoma have focused on the prognostic factors for survival. Thus, there is an unmet need for sufficient studies on visual prognosis, which significantly affects the quality of life of patients with VRL. In this study conducted at a tertiary referral center, we evaluated the clinical features of VRL and prognostic factors for vision loss in patients with VRL.

## Results

A total of 113 eyes of 64 patients were evaluated and followed up for 35.4 ± 25.8 months. The baseline demographic and disease characteristics of the study population are shown in Table 1. The mean age of the patients was 64.2 ± 10.9 years, and 34 (53.1%) patients were female. Among these, 49 (76.6%) showed bilateral ocular involvement. The most common histological diagnosis was diffuse large B-cell type lymphoma (70.3%). At diagnosis, 36 (56.3%), 17 (26.6%), and 11 (17.2%) patients had primary, secondary, and concurrent VRL, respectively. Among the 36 patients with primary VRL, 14, 1, and 1 developed CNS lymphoma, nasal cavity lymphoma, and testicular lymphoma, respectively, during the follow-up period. In patients with secondary VRL, the mean duration between initial CNS lymphoma diagnosis and VRL diagnosis was 19.5 ± 19.8 (range, 2–72) months.

**Table 1:** Baseline demographics and disease characteristics of patients with vitreoretinal lymphoma.

Patient characteristics	Value
Follow-up period (months)	35.4 ± 25.8
Age, years	64.2 ± 10.9
Sex, n (%)	
Female	34 (53.1%)
Male	30 (46.9%)
Bilaterality, n (%)	49 (76.6%)
Subtype	
Primary	20 (31.2%)
Secondary	17 (26.6%)
Ocular to CNS	16 (25.0%)
Concurrent	11 (17.2%)
Months from initial CNSL diagnosis to VRL (months)	19.5 ± 19.8 (range, 2–72 months)
Histology	
DLBCL	45 (70.3%)
NK/T cell	1 (1.6%)
Lymphoplasmacytic	0
Unspecified	18 (28.1%)
Death during follow-up, n (%)	11 (17.2%)
Recurrence of intraocular lymphoma, n (%)	14 (21.9%)
Time interval to recurrence (months)	26.4 ± 16.9 (range, 8–46 months)

Data are expressed as mean ± standard deviation (95% confidence interval).

*CNS* central nervous system, *CNSL* CNS lymphoma, *DLBCL* diffuse large B-cell lymphoma.

The baseline ocular characteristics of eyes with VRL are shown in Table 2. The mean logarithm of the minimal angle of resolution (logMAR) best-corrected visual acuity (BCVA) was 0.44 ± 0.28. Among our study participants, the most common clinical characteristic of VRL was vitreous opacity, observed in 76 (67.3%) eyes. Other clinical characteristics of VRL included retinal/subretinal infiltration (41.6%), anterior-chamber cellular reaction (18.6%), exudative retinal detachment (3.5%), optic nerve hemorrhage/edema (5.3%), and optic-nerve infiltration (1.8%). In our study, 64 (56.6%) eyes were diagnosed based on cytohistopathologic evaluation of vitreous specimens acquired from diagnostic pars plana vitrectomy, and 41 (36.3%) patients were diagnosed based on cytokine analysis, including interleukin (IL)-10, IL-6, and IL-10/IL-6 ratio (Supplementary figure). Other diagnostic modalities included brain and non-CNS tissue biopsies (7.1%).

**Table 2:** Baseline ocular findings of patients with vitreoretinal lymphoma.

Patient characteristics	Value
Best-corrected visual acuity, logMAR	0.44 ± 0.28
Intraocular pressure, mmHg	15.1 ± 4.7
Anterior chamber cell grade	0.41 ± 0.54
Vitreous cell grade	0.80 ± 0.68
Presence of clinical features	
Anterior chamber cellular reaction	21 (18.6%)
Vitreous opacity	76 (67.3%)
Exudative retinal detachment	4 (3.5%)
Retinal/subretinal infiltration	47 (41.6%)
One quadrant	15 (13.3%)
Two quadrants	12 (10.6%)
Three quadrants	3 (2.7%)
Four quadrants	6 (5.3%)
Involving macula	11 (9.7%)
Optic nerve infiltration	2 (1.8%)
Optic nerve haemorrhage/oedema	6 (5.3%)
Diagnosis tool	
Cytology	64 (56.6%)
Cytokine analysis	41 (36.3%)
Others	8 (7.1%)
Cytokine assay in aqueous humour	
Number of data available	58/113
IL-10 (pg/mL)	4504.5 ± 5844.4
IL-6 (pg/mL)	126.4 ± 345.8
IL-10/IL-6 ratio	222.3 ± 506.7

Data are expressed as mean ± standard deviation (95% confidence interval).

The treatment strategies for VRL are summarized in Table 3. All eyes with VRL were treated with intraocular MTX injections. Pars plana vitrectomy was performed to acquire vitreous specimens and remove the vitreous opacity. The mean duration of intravitreal MTX injection was 9.9 ± 3.8 months, with an average of 17.1 ± 5.5 injections. Toxic corneal epitheliopathy after intravitreal MTX was observed in 38 (33.6%) eyes after a mean of 6.8 ± 2.0 injections, and temporary cessation of intravitreal MTX was required in these cases. Systemic treatment was combined with local therapy for CNS lymphoma or in selected cases of isolated VRL, according to the neuro-oncologist’s recommendations. Systemic treatment included systemic chemotherapy (66.4%), external beam radiotherapy (29.2%), and other concurrent systemic treatments (21.2%), such as intrathecal chemotherapy, stereotactic biopsy, and autologous peripheral-blood stem-cell transplantation.

**Table 3:** Systemic and local treatment strategies for vitreoretinal lymphoma.

Treatment strategies	N (%)
Systemic treatment	
Systemic chemotherapy	75 (66.4%)
External beam radiotherapy	33 (29.2%)
Other concurrent systemic treatment	24 (21.2%)
Local treatment	
Pars plana vitrectomy	56 (49.6%)
Intravitreal methotrexate injection	113 (100%)
Duration of intravitreal methotrexate (months)	9.9 ± 3.8
Total number of intravitreal methotrexate	17.1 ± 5.5

Data are expressed as mean ± standard deviation (95% confidence interval).

Improvements in the clinical parameters according to treatment are shown in Table 4. The mean BCVA improved from 0.44 ± 0.28 logMAR at diagnosis to 0.33 ± 0.29 logMAR 1 month after treatment initiation. Vision improved significantly after treatment (final mean BCVA, 0.24 ± 0.21 logMAR; *P* = 0.031). The proportion of eyes with vitreous opacity also decreased significantly (*P* = < 0.001). Cytokine analysis showed that the IL-10 level and IL-10/IL-6 ratio significantly decreased 1 month after treatment initiation and > 6 months after treatment (*P* = 0.010, *P* = 0.048, and *P* = 0.027, respectively).

**Table 4:** Clinical improvement according to treatment.

	Baseline	One month after treatment initiation	Final visit	*P* value
Best-corrected visual acuity, logMAR	0.44 ± 0.28	0.33 ± 0.29	0.24 ± 0.21	.031
Vitreous opacity, n (%)	76 (67.3%)	34 (30.1%)	7 (6.2%)	< .001
Cytokine assay				
IL-10 (pg/mL)	4504.5 ± 5844.4	250.2 ± 58.1	58.1 ± 96.5	.010
IL-6 (pg/mL)	126.4 ± 345.8	61.5 ± 151.7	55.2 ± 92.3	.048
IL-10/IL-6 ratio	222.3 ± 506.7	8.1 ± 22.2	2.6 ± 3.0	.027

Data are expressed as mean ± standard deviation (95% confidence interval).

In this study, significant vision loss was defined as a final BCVA of ≥ 0.5 logMAR. In the univariate analysis, baseline BCVA (*P* = 0.014), exudative retinal detachment (*P* = 0.011), and retinal/subretinal infiltration (*P* = 0.019) were significantly correlated with significant vision loss. Statistically significant variables were included in the multivariate analysis using backward stepwise selection methods. Baseline BCVA (β = 0.234, *P* = 0.025) and retinal/subretinal infiltration (β = 0.108, *P* = 0.005) were significantly correlated with significant vision loss. Concurrent CNS lymphoma, response to local treatment at 1 month, and treatment strategy showed no correlation with significant vision loss (Table 5).

**Table 5:** Univariate and multivariate linear regression analyses to evaluate the predictors of significant vision loss in eyes with vitreoretinal lymphoma following treatment.

	Univariate			Multivariate		
	β	95% CI	*P*	β	95% CI	*P*
Age	0.005	− 0.007, 0.019	.251			
Sex	0.141	0.415, 0.275	.637			
Concurrence with CNSL	0.094	0.323, 0.138	.365			
Baseline BCVA	0.329	0.326, 1.934	.014	0.234	0.078, 1.042	.025
Anterior chamber cell	0.138	0.566, 0.112	.152			
Vitreous cell	0.110	0.319, 0.220	.667			
Vitreous opacity	0.181	0.058, 0.944	.052	0.179	0.314, 0.430	.748
Exudative retinal detachment	0.410	0.460, 2.467	.011	0.428	0.837, 0.930	.615
Retinal/subretinal infiltration	0.172	0.126, 0.966	.019	0.108	0.101, 0.533	.005
Optic nerve infiltration	0.075	0.200, 0.167	.831			
Baseline IL-10	0.0005	0.0001, 0.0002	.178			
Baseline IL-6	0.003	0.013, 0.0002	.056	0.0001	0.0002, 0.0004	.446
Baseline IL-10/IL-6 ratio	0.0002	0.001, 0.0004	.055	0.0001	0.0003, 0.0001	.455
Treatment response at 1 month						
Change in BCVA	0.686	1.331, 1.831	.725			
Change in IL-10/IL-6 ratio	629.7	2384.0, 520.0	.177			
Systemic treatment strategy						
Systemic chemotherapy	0.237	0.662, 0.496	.738			
Systemic radiotherapy	0.171	0.232, 0.603	.319			

*BCVA* best-corrected visual acuity, *CNSL* CNS lymphoma.

## Discussion

In this study, we evaluated the clinical features and treatment outcomes of VRL, especially focusing on the prognostic factors for significant vision loss after treatment. Our study showed that baseline BCVA and retinal/subretinal infiltration were significant predictors of vision loss after treatment for VRL.

The diagnosis of VRL is challenging and usually delayed because of the diverse clinical presentations, small volume of intraocular fluid available for testing, and initial response to steroid therapy^11^. Common clinical features of VRL include large, non-clumped vitreous cells, retinal infiltration, sub-retinal pigment epithelium (RPE) deposits, and RPE undulation^12,13^. When clinically suspected, a confirmative diagnostic test should be conducted. In the past years, the gold standard for diagnosis was the detection of malignant lymphoid cells in vitreous samples^14,15^. Immunohistochemistry, cytokine concentration analysis (IL-10/IL-6 ratio), flow cytometry, and polymerase chain reaction tests to detect immunoglobulin heavy chains or T-cell receptor gene rearrangement, respectively, may also support the diagnosis of VRL^15,16^. In our study, cytological analysis of vitreous specimens (56.6%) and cytokine analysis (36.3%) were the most commonly used methods to confirm the diagnosis of VRL.

In this report, we present the prognostic factors for vision loss in patients with VRL. In our study, the overall visual outcome was relatively good; the baseline mean BCVA was 0.44 ± 0.28, and the final mean BCVA was 0.24 ± 0.21. Our findings are consistent with the overall good visual outcomes in patients with VRL reported in the literature. However, few studies have evaluated factors predicting visual outcomes following treatment. Cho et al.^7^ evaluated 53 patients with VRL and reported good vision after treatment; the mean final BCVA was 1.047 ± 1.068 after follow-up for 32.5 ± 24.6 months. The final visual acuity did not differ in the primary, secondary, and concurrent VRL groups. Habot-Wilner et al.^17^ reported that in 129 patients with VRL, 62.8% of the eyes improved or maintained their initial vision after treatment. Dalvin et al.^18^ reported that eyes with sub-RPE infiltration were more likely to have a poor final visual acuity (< 20/200) than eyes without sub-RPE infiltration. This result is consistent with our findings. In our study, baseline BCVA, exudative retinal detachment, and retinal/subretinal infiltration were significant predictors of significant vision loss (final BCVA < 0.4). In our study population, exudative retinal detachment and retinal/subretinal infiltration were observed in 3.5% and 41.6% of the eyes, respectively. A worse visual outcome after treatment for VRL with subretinal infiltration and exudative retinal detachment is considered to result in RPE atrophy with secondary photoreceptor dysfunction^18^. Alessandro et al.^19^ reported that 52% (41 eyes of 79 eyes) of their VRL cohort showed chorioretinal atrophy (CRA) and the eyes with CRA had a significantly worse vision. Major risk factors of developing CRA were retinal infiltrates, vertical hyperreflective lesions, and macular involvement.

The optimal treatment strategy for VRL remains controversial and nonuniform. Therapeutic options range from local to systemic treatment, including chemotherapy and radiation. Local ocular treatment includes intravitreal methotrexate (MTX) or rituximab injection or external-beam radiotherapy^14,17,20,21^. Systemic management of isolated VRL and PCNSL with ocular involvement should be performed in collaboration with ophthalmologists and other experts, such as neuro-oncologists, hematologists, and radiologists^22^.

Intravitreal MTX injection remains the gold standard for the management of VRL and is usually effective in controlling intraocular inflammation^9,21,23–25^. However, the necessity for additional systemic treatment for patients with isolated VRL without CNS lymphoma is unclear. In our study, physicians did not administer additional systemic chemotherapy to most patients without CNS involvement (17/20, 85%). Only 3 patients with primary VRL (3/20, 15%) were treated with systemic high-dose MTX chemotherapy in addition to intravitreal MTX injection. Recent studies have shown that in patients with isolated VRL, supplementary systemic chemotherapy or radiotherapy has no additional benefit in terms of survival rate, time to relapse or progression, or mean time to CNS lymphoma development. In 2011, the International Primary Central Nervous System Lymphoma Collaborative Group published treatment guidelines for primary VRL^14^. They suggested that the addition of systemic chemotherapy to intravitreal therapy should be considered in cases of bilateral involvement. However, if VRL occurs in only one eye and there is no CNS or systemic involvement, local treatment alone is sufficient. Local treatment includes intravitreal MTX, intravitreal rituximab, or external-beam radiotherapy to the eye.

Our study had some limitations that must be considered when interpreting our findings. First, owing to the retrospective design, the results were susceptible to ascertainment bias, and the treatment strategies were not standardized. Second, in patients with the more aggressive subtype of lymphoma, the long-term visual prognosis is difficult to determine because of the short survival period and high mortality. Third, the results may be confounded by a lack of data on relevant variables, such limited multimodal imaging findings and combinations of treatment strategies.

In summary, we report a relatively good and well-maintained overall visual outcome at > 35 months. Baseline BCVA and retinal/subretinal infiltration were significant predictors of vision loss after treatment with VRL.

## Methods

### Study population

This retrospective longitudinal study included consecutive patients with VRL treated between January 2006 and December 2020 at Seoul St. Mary’s Hospital. The inclusion criteria were a diagnosis of VRL and age > 18 years.

The exclusion criteria were (1) patients with a history of treatment for uveitis, including intravitreal injections or vitreoretinal surgery; (2) patients whose uveitis was unlikely to be related to VRL; (3) patients with concomitant ocular diseases, including retinal vein occlusion, glaucoma, diabetic retinopathy, or epiretinal membrane; (4) patients unable to undergo ophthalmic examination or treatment due to deterioration of their general condition; and (5) patients with recent serious or chronic infection (such as hepatitis B, hepatitis C, human immunodeficiency virus infection, or sepsis). The study design followed the principles of the Declaration of Helsinki, and all protocols were approved by the Institutional Review Board of the Catholic University of Korea. The institutional review board waived the requirement for informed consent because of the retrospective nature of this study.

The diagnosis of VRL was confirmed by combining typical clinical features with cytology and cytokine analysis of the anterior chamber and vitreous. Cytokine levels, including IL-10 and IL-6, were measured using a bead-based assay on a LUMINEX MAGPIX system (Luminex Corp., Austin, TX, USA). An elevated IL-10 level or IL-10-to-IL-6 ratio > 1 is indicative of VRL^26,27^. All patients diagnosed with VRL were referred to the hematology and neuro-oncology departments for systemic evaluation and treatment. The cohort included patients with primary VRL, secondary VRL (vitreoretinal involvement at relapse), and VRL concurrent with CNS or systemic lymphoma.

### Patient evaluation and treatment

We collected and reviewed clinical and demographic data, including age, sex, follow-up duration, medical history, and history of ocular and systemic treatments. During follow-up, comprehensive ophthalmic evaluations, including BCVA, slit-lamp examination, tonometry, and dilated fundus examination, were performed at each visit. BCVA was converted to logMAR for statistical analyses. Eyes underwent either spectral-domain optical coherence tomography (OCT) (Spectralis HRA OCT; Heidelberg Engineering, Heidelberg, Germany) or swept-source OCT (DRI-OCT; Topcon Corp, Tokyo, Japan). For each patient, the same OCT device was used throughout the follow-up period. The presence of clinical features, such as anterior-chamber cells, keratic precipitates, vitreous cells, retinal/subretinal infiltration, exudative retinal detachment, and optic-nerve infiltration/hemorrhage/edema, was evaluated.

### Statistical analyses

An exploratory analysis was performed for all variables. Categorical data are expressed as absolute numbers and continuous data as mean ± standard deviation (95% confidence interval). We performed a linear mixed model to compare improvements in clinical parameters, including BCVA, vitreous opacity, and values of cytokine-assay parameters after treatment. Univariate and multivariate linear regression analyses were performed to evaluate predictors of significant vision loss following treatment in eyes with VRL. In this study, significant vision loss was defined as a final BCVA of ≥ 0.5 logMAR. All statistical analyses were performed using the Statistical Package for the Social Sciences for Windows (version 24; IBM, Armonk, New York, USA). Statistical significance was set at* P* < 0.05.

## Supplementary Information

Supplementary Figures.

## Data Availability

The authors confirm that the data supporting the findings of this study are available within the article. Raw data that support the findings of this study are available from the corresponding author, upon reasonable request.
