# Supplementary figures and images for: Clinical features predictive of vision loss in patients with vitreoretinal lymphoma: a single tertiary center experience
Source: Sci Rep. 2023 Mar 18;13:4478. doi: 10.1038/s41598-023-31414-0 (PMC10024690; doi:10.1038/s41598-023-31414-0)

**Supplementary Figure.** Baseline distribution of IL10, IL6 and IL 10/6 ratio


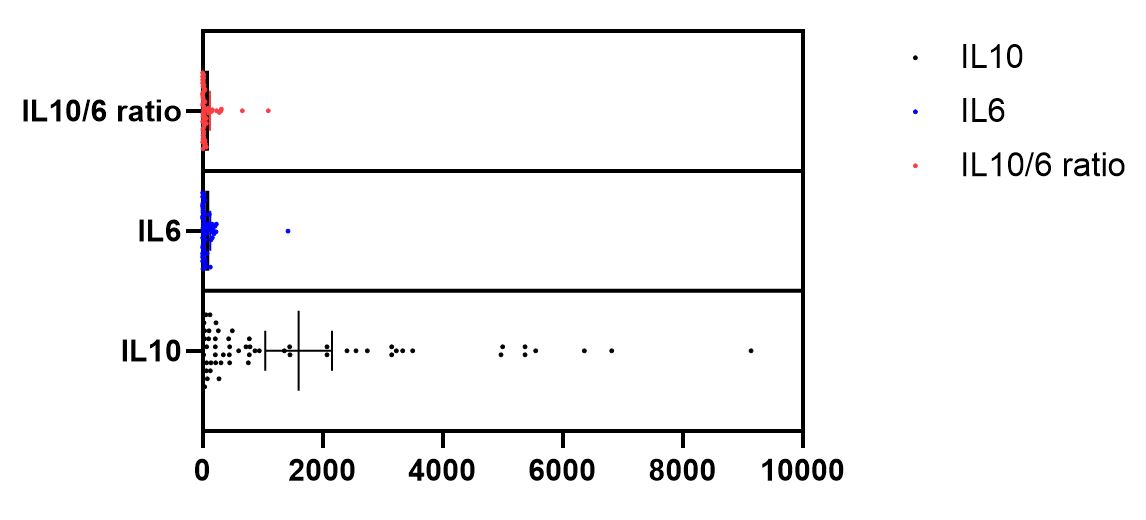

Supplement: Supplementary file 1 — Supplementary Figures. [file 41598_2023_31414_MOESM1_ESM.docx]
